# Development of a Low-Cost, Modular Muscle–Computer Interface for At-Home Telerehabilitation for Chronic Stroke

**DOI:** 10.3390/s21051806

**Published:** 2021-03-05

**Authors:** Octavio Marin-Pardo, Coralie Phanord, Miranda Rennie Donnelly, Christopher M. Laine, Sook-Lei Liew

**Affiliations:** 1 Department of Biomedical Engineering, University of Southern California, Los Angeles, CA 900089, USA; marinpar@usc.edu; 2 Chan Division of Occupational Science and Occupational Therapy, University of Southern California, Los Angeles, CA 900089, USA; phanord@usc.edu (C.P.); mrennie@usc.edu (M.R.D.); christopher.laine@chan.usc.edu (C.M.L.)

**Keywords:** biofeedback, stroke, telerehabilitation, electromyography, human-computer interface

## Abstract

Stroke is a leading cause of long-term disability in the United States. Recent studies have shown that high doses of repeated task-specific practice can be effective at improving upper-limb function at the chronic stage. Providing at-home telerehabilitation services with therapist supervision may allow higher dose interventions targeted to this population. Additionally, muscle biofeedback to train patients to avoid unwanted simultaneous activation of antagonist muscles (co-contractions) may be incorporated into telerehabilitation technologies to improve motor control. Here, we present the development and feasibility of a low-cost, portable, telerehabilitation biofeedback system called Tele-REINVENT. We describe our modular electromyography acquisition, processing, and feedback algorithms to train differentiated muscle control during at-home therapist-guided sessions. Additionally, we evaluated the performance of low-cost sensors for our training task with two healthy individuals. Finally, we present the results of a case study with a stroke survivor who used the system for 40 sessions over 10 weeks of training. In line with our previous research, our results suggest that using low-cost sensors provides similar results to those using research-grade sensors for low forces during an isometric task. Our preliminary case study data with one patient with stroke also suggest that our system is feasible, safe, and enjoyable to use during 10 weeks of biofeedback training, and that improvements in differentiated muscle activity during volitional movement attempt may be induced during a 10-week period. Our data provide support for using low-cost technology for individuated muscle training to reduce unintended coactivation during supervised and unsupervised home-based telerehabilitation for clinical populations, and suggest this approach is safe and feasible. Future work with larger study populations may expand on the development of meaningful and personalized chronic stroke rehabilitation.

## 1. Introduction

Stroke is a leading cause of long-term disability in the United States with almost 800,000 people experiencing a new or recurrent stroke each year [1]. While motor recovery was thought to plateau by the chronic stage after stroke (more than 6 months after the vascular incident), more recent studies have shown that improvement of upper limb function is possible at the chronic stage [2,3]. Recent research suggests that high dose interventions of repeated task-specific practice are effective at inducing significant positive outcomes in this population [3,4,5,6]. However, due to the time and physical constraints of many therapy sessions, common in-clinic interventions only provide on average 32 repetitions of functional upper extremity movements per session [7]. Providing at-home telerehabilitation services with therapist supervision and input is a potential solution to allow clinicians to deliver quality, higher dose interventions. Recent studies suggest that telerehabilitation for stroke rehabilitation is feasible and as effective as in-person therapy [8,9].

One effective method for improving upper limb function, which could be combined with telerehabilitation, is the reinforcement of muscle activity using electromyography (EMG) biofeedback. Muscle biofeedback has been shown to reduce spasticity and improve post-stroke arm function, motor control, muscle activity, and strength [10,11]. Specifically, previous research has shown that biofeedback training to avoid unintended simultaneous activation of antagonist muscle groups may be particularly beneficial for reducing unnecessary co-contractions that impede functional motor control [12,13]. However, further research is required to address remaining fundamental questions in biofeedback investigations—for example, what is the required intensity and dosage to significantly improve long-term outcomes?

Recently, portable systems have been developed for at-home use to improve accessibility and training time with EMG biofeedback [13,14]. However, proper implementation of home-based EMG biofeedback is critical to prevent low participant adherence, avoid high costs, and account for limitations in terms of required physical space, time, and technical literacy [14,15,16,17,18]. Specifically, it has been suggested that the ability to track patient progress in real-time and the continued involvement of a clinician in the intervention are key factors that could improve patient motivation and adherence to at-home rehabilitation programs [9,17].

To address these needs, we developed and tested a low-cost, portable telerehabilitation biofeedback system called Tele-REINVENT. Tele-REINVENT builds upon our previous work in which we developed and tested a system (REINVENT) that could provide biofeedback of brain or muscle activity on a computer screen or in immersive virtual reality (VR) with a head-mounted display (HMD) in a laboratory or clinic setting [19,20]. Tele-REINVENT uses the same modular platform and incorporates a telerehabilitation component for live video and audio conferencing with a clinician who meets regularly with the participant to monitor progress. The clinician can also provide technical support, ensure the electrodes are placed correctly, and monitor EMG signals in real-time to ensure adequate signal quality. In addition, Tele-REINVENT uses a portable laptop with commercial low-cost EMG sensors for greater affordability and accessibility in the home environment. Lastly, Tele-REINVENT has new gamified elements to encourage greater engagement, motivation, and adherence to a home-based program. Overall, we aimed to incorporate benefits from both literature on telerehabilitation and EMG biofeedback into our current system.

In this paper, we present a detailed description of the Tele-REINVENT system. Additionally, we provide a validation example with two healthy individuals showing that, for our purposes, the performance of the low-cost sensors can be considered comparable to that of research-grade sensors. Finally, we present the feasibility and results of a case study with a chronic stroke survivor who tested the system for 40 sessions over 10 weeks.

## 2. Materials and Methods

### 2.1. Participants

To confirm that low-cost EMG sensors produced valid and appropriate measurements, for our purposes, as compared with research-grade equipment, we compared measurements collected from 2 healthy male right-handed participants (ages 28 and 37 years old) using both systems.

For the case study, we recruited a 67-year-old male stroke survivor, 11 years after stroke onset, to test our developed system for 10 weeks. The participant had upper extremity hemiparesis, was not taking anti-spasticity medication, had no receptive aphasia, had corrected vision, and did not have a secondary neurological disease. The participant had less than 15 degrees of active wrist or finger extension in the more impaired hand and was unable to grasp and release a ball unassisted.

Protocols were approved by the University of Southern California Health Sciences Campus Institutional Review Board (IRB) and written informed consent was obtained from all participants in accordance with the Declaration of Helsinki.

### 2.2. System Architecture

**Figure 1:**
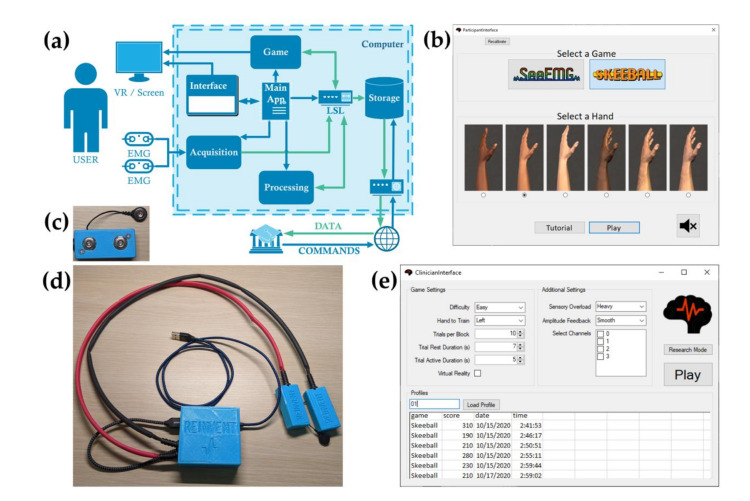
Tele-REINVENT architecture, prototype, and interfaces. (**a**) Software architecture consisting of an acquisition client for electromyography (EMG), signal processing scripts, and a game engine for feedback visualization. Each module is managed by the main application and all data is internally streamed and stored using the Labstreaming Layer (LSL) protocol. This architecture supports both remote data retrieval and updates. Green arrows represent data and blue arrows configurations and commands. (**b**) Participant graphical interface. Here, the participant can select the game to play and set user configurations accordingly. (**c**) Case and Myoware sensor with connectors for disposable EMG electrodes. (**d**) Tele-REINVENT prototype consisting of 2 Myoware EMG sensors, a Teensy 4.0 development board, and a USB isolator enclosed in 3D-printed cases. (**e**) Clinician graphical interface. Here, the clinician can set up configurations for the biofeedback game, such as number of trials, trial duration, and difficulty. Additionally, the interface can show data from previously recorded sessions.

Tele-REINVENT is a portable stroke telerehabilitation system for at-home, therapist-driven, personalized, and gamified training. We designed the system to acquire reliable EMG signals and provide realistic feedback as game control. We designed and developed hardware and software that allowed us to acquire, process, and store the participant’s EMG signals on a laptop computer (Razer Blade RZ09-01953; Operating System: Windows, Processor: Intel Core i7 7700, RAM: 16 GB; Razer Inc., Irvine, CA, USA). Furthermore, the architecture of the system allows for remote update, control, and data retrieval. Figure 1a shows the overall architecture of the system. Briefly, a C# application developed in Visual Studio (Community 2019, Microsoft, Redmond, WA, USA) controlled the information flow between the system modules and graphical user interfaces (GUI), while the Labstreaming Layer protocol (LSL) [21] transmitted data between modules for processing, game interaction, and storage. Importantly, connecting modules through the LSL network provides an architecture that allows for the functional independence of each component. This is advantageous for continued development since different configurations of sensors, processing pipelines, and environments can be implemented without necessitating updates to other modules. Specifically, while we only tested EMG biofeedback on a laptop screen in the current study, the system builds on the modular architecture we developed in previous work, allowing for the input of electroencephalography or movement data, and visual output to either a laptop screen or HMD-VR system as well as proprioceptive feedback via handheld controllers [19,20]. Additionally, we incorporated a NeuroPype script (Intheon, San Diego, CA, USA) for real-time visualization of the digitized EMG signals.

We designed Tele-REINVENT with several GUI configuration screens to accommodate different user needs and allow for user-friendly set-up depending on the user’s role as a participant, clinician, or researcher. In this way, Tele-REINVENT can be used as a system for stroke survivors to use on their own at home, with minimal interaction and configuration required for set up, as shown in Figure 1b. Alternatively, as tested here, we also believe that effective training requires a trained clinician to specify parameters based on the participant’s profile and, thus, we developed a second GUI specifically for clinicians to use and customize, shown in Figure 1e. This interface also helps to quantify clinical data for the clinician to monitor progress. Finally, researchers may also desire to use this system and tune more specific parameters to test different hypotheses; there is a third GUI with more detailed parameters to configure.

### 2.3. Acquisition

We used a Teensy 4.0 development board (PJRC.COM LLC, Sherwood, OR, USA) and a pair of Myoware muscle sensors (Advancer Technologies LLC, Raleigh, NC, USA) to acquire signals from the wrist extensor and flexor muscle groups. We designed and manufactured 3D-printed cases to enclose all hardware components to provide electrical insulation and improve durability. First, each Myoware sensor amplified the signal registered between a pair of differential electrodes. Then, the Teensy board digitized the signals and sent them to the computer through a USB cable via serial communication. Finally, a C# application connected the board with the computer and streamed the signals to a local network using the LSL protocol. All scripts were custom made using Visual Studio and Arduino Software (Arduino AG). This configuration allowed us to acquire up to 4 channels of EMG signals at 2000 Hz with 12-bit ADC resolution. Importantly, the Myoware sensors are not capable of in-board filtering; raw EMG signals are only differentially amplified. The Myoware board was designed to be used with microcontrollers that require positive voltages for analogic acquisition, e.g., an Arduino or a Teensy board. Thus, Myoware centers the amplified signal around half the voltage used to power it, that is, about 1.65 V when powering the Myoware with 3.3 V. This is done by the electronics of the board as part of the signal conditioning.

### 2.4. EMG Signal Processing and Biofeedback

**Figure 2:**
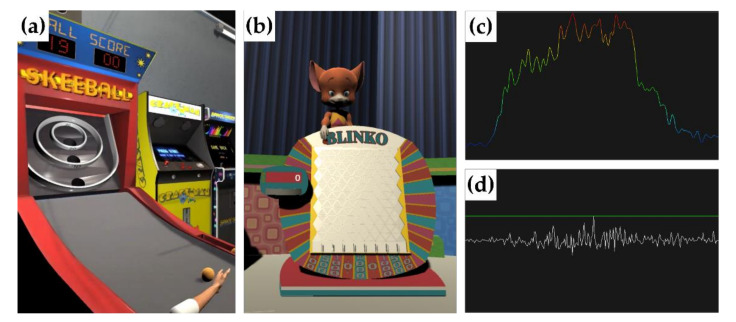
Examples of EMG biofeedback games. (**a**) *SkeeBall*: adequate extensor ratio muscle activity translates as movement of the arm shooting the ball to different targets. (**b**) *Blinko*: adequate extensor ratio muscle activity translates as movement of the character left and right, who then drops a chip to win points. (**c**) *SeeEMG*: smooth EMG is mapped to show high amplitudes in red and low amplitudes in blue. A dynamic range to scale amplitudes can be modified by the clinician. (**d**) *SeeEMG*: down-sampled rectified EMG. The green horizontal line represents the maximum value reached in that session.

We developed custom scripts in Matlab (R2020a, The Mathworks, Natick, MA, USA) to process EMG signals in real-time and use them for game control. Digitized signals were filtered, rectified, and normalized to a prerecorded maximum grip. The filter we implemented also removes the DC offset that may be present in the digitized signal. Thus, a value of normalized activity close to 0 corresponds to no volitional activity registered by the sensor and a value close to 1 represents an amplitude similar to that seen during the attempted grip. The clinician or researcher can modify the specifics of the processing algorithms based on the study needs. Each game was developed in the Unity game engine (v2019.3.12f1, Unity Technologies, San Francisco, CA, USA) to provide feedback in the form of different games and stream game interactions (e.g., current score and trial number) through the LSL protocol. Games currently in the Tele-REINVENT suite are described below:

- **SeeEMG**—The purpose of this game is to provide quick visual feedback of real-time extensor and flexor muscle activity as independent continuous streams. After applying the signal processing steps described above, EMG signals are plotted in each frame as a continuous line for each of the muscles. The participant is allowed to select one of two configurations. Option 1 displays EMG smoothed with a 250-ms Hann window, down-sampled to the refresh-rate of the screen. This option uses a heatmap color scale to map high amplitudes in red and low amplitudes in blue (Figure 2c). The default range sets “high” as the same amplitude as the maximum grip used to calibrate the system, and “low” as the lowest amount of activity (typically, none) recorded during calibration. This range can be dynamically adjusted by the clinician based on the average amplitude of the signals to ensure the whole range of colors is rendered. Option 2 displays rectified EMG, down-sampled to the refresh rate of the screen, as white lines. A dynamic green horizontal line is also plotted to represent the maximum amplitude reached during that session (Figure 2d).
- **SkeeBall**—The purpose of this game is to improve wrist extensor activity and control while reducing abnormal coactivation of wrist flexors during active extension attempts. In this game, we use the ratio of activity from the wrist extensors and flexors to move a ball to different targets. First, we apply the signal processing steps described above. Then, we calculate an extensor ratio (ER) value (shown in Equation (1)), defined as the sum of the mean extensor activity divided by the sum of the mean activity from extensor and flexor muscles.

(1) $$\mathrm{ER}=\frac{\mathrm{extensor}}{\mathrm{extensor}+\mathrm{flexor}}$$

ER values closer to 1 indicate more individuated extension, closer to 0.5 more coactivation, and closer to 0 more individuated flexion. Previous research has suggested different effective methods to calculate the contribution of antagonist activity in different movements and muscle groups [12,22,23,24]. We selected the current approach for its simplicity, low computational requirements, and its ability to capture the relationship of two antagonistic muscles. As noted above and mentioned in our previous work [20], we expect that using this ratio is advantageous to encourage wrist extension without simultaneous unintended wrist flexion. For each trial, a score is assigned as a function of the ER value and a probability likelihood as shown in Table 1. That is, an ER value of 0.7 would result in 20 points in 60% of trials and 30 points in 40% of trials. Calculated point values trigger the corresponding animation of the hand hitting the ball and the ball moving to the appropriate ring (Figure 2a). At the end of the game, the final score is calculated as the cumulative points for each trial. Importantly, the probability values shown in Table 1 are only used to determine the score of the trial. That is, a calculated value of individuated extension (ER > 0.5) will always be positively reinforced, providing higher scores to higher individuation values.

- **Blinko**—The purpose of this game is to improve both wrist extensor and wrist flexor activity while reducing abnormal coactivation. Similar to *SkeeBall*, we use the ratio of activity from the wrist extensors and flexors, the extensor ratio (ER), to move a round flat disc, or chip, across the top of a board. The board was modeled after the popular gameshow chance game *Plinko*, as shown in Figure 2b. The board consists of rows of pegs, with each row offset from the one above it. At the bottom of the board, there are nine slots that represent points. These are labeled as follows: $100, $500, $1000, $0, $10,000, $0, $1000, $500 and $100. When a chip is dropped at a slot between two pegs at the top of the board, the chip is diverted by the slots below it. Thus, the chip may take any number of paths to the bottom and land in any of the nine slots. For each trial, the player attempts to move the chip right with wrist extension or left with wrist flexion. Then, the player drops the chip and the points assigned correspond with the bottom slot where the chip landed. At the end of the game, the final score is calculated as the cumulative points for each trial.

### 2.5. Example of a Tele-REINVENT Telerehabilitation Session with a Clinician

**Figure 3:**
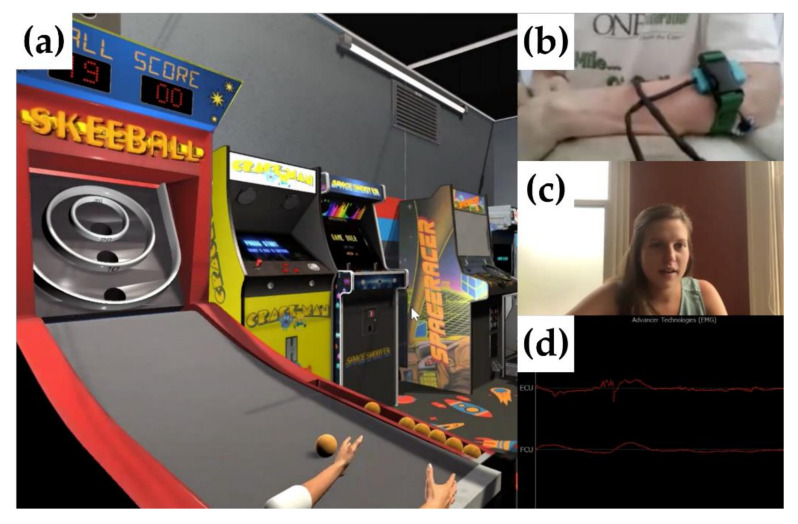
Feedback displayed during a session guided by an occupational therapist. (**a**) *SkeeBall* game rendered on the computer screen. (**b**) View from the laptop camera of the participant’s arm with electrode placements. (**c**) Occupational therapist guiding the session. (**d**) Real-time visualization of the EMG signals.

Before starting the telerehabilitation sessions, a trained clinician administers a series of behavioral assessments to quantify the participant’s baseline level of impairment to determine training parameters, e.g., training duration, rest intervals, and the number of repetitions per game. These parameters are set by the clinician or researcher and used for remote training sessions but can be modified on a regular basis as sessions advance. Then, the participant undergoes a detailed orientation showing them step-by-step how to use the system and how to properly place the sensors. Alternatively, if it is unfeasible to do all procedures in person (e.g., due to logistics or social distancing protocols), the orientation can also be performed using the incorporated video conferencing application (Zoom Video Communications, Inc., San Jose, CA, USA). Training sessions can be done by the participant alone or remotely monitored by the clinician or researcher. Figure 3 shows an example of a guided session in which the screen can be configured to view real-time performance of *SkeeBall* game, the participant’s electrode placement and body movements, the clinician, and real-time EMG signals viewed all at once. Video meetings with the occupational therapist can take place as often as needed (e.g., in our case study described below, they were performed daily for the first week, and afterward on a bi-weekly basis or if there were technical problems with the system). The participant and clinician also communicated every other day via email. As shown in previous studies, regular contact with the clinician is critical for maintaining participant engagement and adherence [9].

Our Tele-REINVENT system consists of a laptop computer with all necessary programs preloaded, configured, and displayed in an easy-to-use manner, a pair of EMG sensors with the enclosed acquisition board, and a package of disposable electrodes. When participants first receive the equipment, they are also given a printed manual of how to use the system. For enhanced simplicity and consistency, each session starts with instructional videos that remind the participant how to start a telerehabilitation session, plug in the acquisition device, and position the sensors over the targeted muscles. Then, a calibration video guides the participant through wrist and hand movements, including gross grasp, wrist extension, and wrist flexion. Upon completion of the recording, a Matlab script filters and rectifies the signals, as described above, and calculates the mean amplitudes during the grip. These values are later used as a calibration to normalize real-time signals. Then, the participant selects which game they will play and sets visual configurations for the game, e.g., color-coded signals or arm model (Figure 1b). Once the participant is ready, the game starts. The number of repetitions per game, rest periods between trials, and the duration of gameplay is determined by the researcher or clinician in collaboration with the participant. These parameters are influenced by fatigue, spasticity, endurance, and other personal factors. Finally, after completion of each training session, de-identified recorded data is securely synchronized to the clinician’s or researcher’s computer via Google Drive (Google, Mountain View, CA, USA).

### 2.6. Signal Quality Validation Example

We present an example in two healthy individuals to validate the use of low-cost Myoware sensors by comparing calculated values of muscle coactivation with those obtained using a research-grade Delsys Trigno Wireless System (Delsys Inc., Natick, MA, USA). Table 2 presents a summary of the specifications for both sensors.

As noted previously, for this analysis we recruited 2 male participants and recorded a series of repeated time-constrained ramp-up and hold isometric grips, similar to what was performed during the Tele-REINVENT tasks. First, we cleaned the skin with isopropyl alcohol and electrodes were placed over the extensor carpi radialis and flexor carpi ulnaris of the dominant hand. Proper positioning was confirmed via palpation and observation of the EMG signals during wrist flexion, extension, ulnar deviation, radial deviation, and gross grip. Then, we simultaneously recorded EMG and exerted force during a 3-s power grip using 2 Trigno Mini sensors and a digital grip dynamometer (Biometrics Ltd., Ladysmith, VA, USA). All signals were digitized with a data acquisition system (USB-6210, National Instruments, Austin, TX, USA) at 1000 Hz and acquired with a custom interface in Matlab.

**Figure 4:**
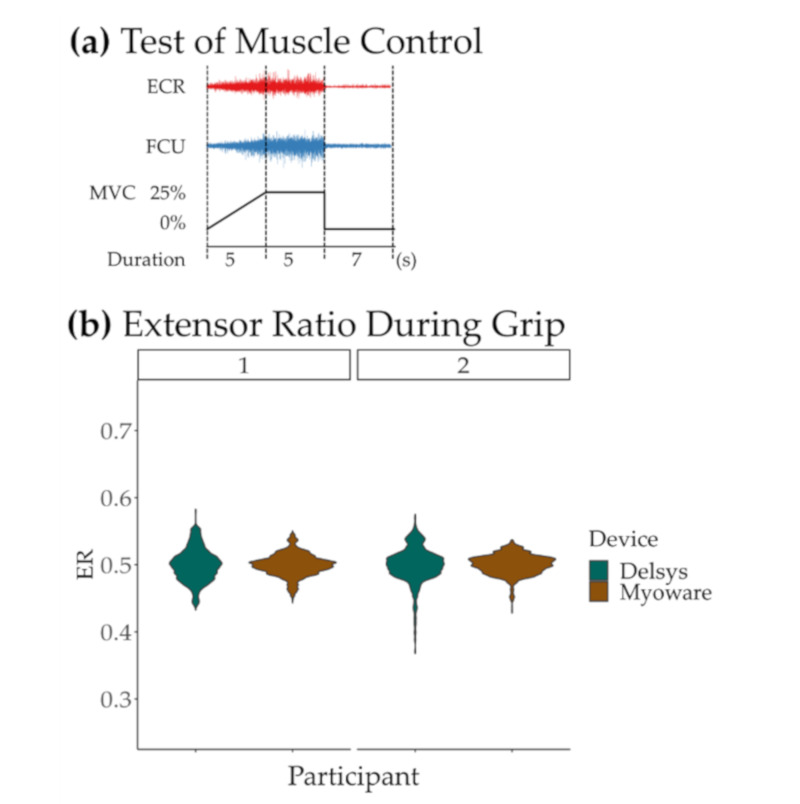
(**a**) Test of muscle control. Two healthy control participants received feedback of their exerted grip force and were asked to follow an amplitude-guided 5-s build-up to a hold level (25% of their maximum voluntary contraction (MVC)) during 5-s epochs, followed by 7 s of rest. The activity of both muscles was analyzed during the ramp-up and hold phases of the task (first two regions marked within dashed lines). (**b**) Calculated extensor ratio values for each time point of the 24 5-s isometric grip holds (12 grip holds with each device) using data acquired from extensor carpi radialis (ECR) and flexor carpi ulnaris (ECU) with Delsys and Myoware for each participant (denoted as 1 and 2). All distributions are centered around 0.5 indicating a similar proportion of muscle activity recorded from both muscles with either device.

To quantify the performance of each sensor, each participant sat in front of a computer screen, their arms rested comfortably on a table and we provided feedback of exerted force during isometric grip holds. We asked the participant to follow the target shown in Figure 4a consisting of 5 s to reach 25% of the previously recorded maximum grip force, 5 s holding the force level, and 7 s of rest. We provided visual feedback with a cursor swiping through the screen from left to right crossing the screen in 17 s and moving up and down according to the current force amplitude until the completion of 12 trials. Finally, we removed the Trigno Mini sensors, placed the Myoware sensors over the same location, and repeated the feedback task. We used the same calibrated grip force for recordings with both sensors.

For offline analysis of both systems, EMG signals were preprocessed in Matlab with a bandpass filter between 150 and 450 Hz, full-wave rectified, and smooth with a moving-average rectangular window of 1s. This filter was chosen to match the preprocessing methods for the patient case study (see below), although using a wider range of 15 and 450 Hz results in no differences. Epochs of the hold phase were extracted to calculate values of normalized ER. Finally, we used paired t-tests to evaluate whether distributions of ER values acquired with the low-cost Myoware sensors were comparable to those acquired with the research-grade Delsys system. We used linear regressions to evaluate the proportion of explained variance between the muscle activity and the corresponding grip force during the ramp-up phase of the trials. We utilized custom scripts in R (version 4.0.3, R Foundation for Statistical Computing, Vienna, Austria) to perform all statistical analyses.

### 2.7. Case Study and Feasibility

We tested the feasibility and preliminary effects of using this system by delivering Tele-REINVENT to a participant with stroke. We asked him to complete 40 sessions that spanned 10 weeks of training. For each session, the participant completed 5 blocks of 20 repetitions using the *SkeeBall* game, described above, for 100 trials per session, which took approximately 1 h per session. It is important to note that it was not possible to perform in-person behavioral assessments before and after use, nor in-person familiarization with the system to avoid unnecessary risks due to ongoing social distancing protocols during the COVID-19 pandemic. Similarly, it was not possible to demonstrate proper sensor placement in-person. All familiarization and verification of sensor placement was performed remotely via videoconferencing with the clinician, who used the real-time EMG signal tracking to assess electrode placement. Therefore, signal quality could still be variable across training sessions. In addition, the participant also actively collaborated on providing user feedback and testing the device during the initial system development for several weeks prior to beginning data collection. Because these sessions were part of familiarization and configuration tunning, and not carefully structured, they are not included in the current analysis.

For real-time online analysis, digitized EMG signals were bandpass filtered between 150 and 450 Hz, rectified, and normalized to the calculated activity during the grip attempt. Then, we calculated ER values to provide positive reinforcement of individuated extension using the SkeeBall game, as described above. Importantly, we selected this higher frequency range to account for possible artifacts induced by motion, crosstalk, and increased susceptibility to environmental noise. Larger contributions from these artifacts were expected due to the lack of in-board filtering of the Myoware sensors and lack of in-person training. Previous research has shown benefits of using high pass frequency filters at the 100 Hz range or above to account for such artifacts and obtain better estimations of force, joint stability, motor unit recruitment, intermuscular coherence, and corticomuscular coherence [25,26,27,28,29,30,31,32,33].

For offline analysis of the recorded sessions, trial data from the game and EMG signals were processed with a custom script in Matlab. First, trial number and EMG signals were interpolated and down-sampled, respectively, to 1000 Hz. Then, signals were processed with the same pipeline as the online data; that is, bandpass filtered between 150 and 450 Hz, full-wave rectified, and normalized to the session’s recorded calibration with values of maximum activity within a 250-ms moving window. Then, values of averaged activity were calculated from the first 2 s of each movement attempt trial. To account for the within-session variability and evaluate how these activity patterns changed over time, we averaged the activity of the 100 trials of each session. Finally, we used Pearson’s correlations to test for significant changes in EMG signals and game performance across 28 training sessions. 12 sessions were excluded from the analysis due to excessive noise in the recording or missing data. We examined session averages for normalized extension activity, flexion activity, and ER with custom scripts in R.

## 3. Results

### 3.1. Signal Quality Validation Example

In this validation example, Myoware and Delsys systems showed no significant differences in ER values during isometric grip holds. Paired t-tests, one for each participant, showed no significant difference in ER values when comparing the two sensors (participant 1: *t* = −0.025, *p* = 0.98; participant 2: *t* = −2.018, *p* = 0.068), as shown in Figure 4b. To ensure the systems do not show a significant divergence within lower forces, we used linear regressions of the normalized activity recorded from each muscle to the measured force during the ramp phase of the trials, shown in Table 3. Across both participants, between 79 and 94% of the variance in force was explained with the normalized EMG signals when these were acquired using the Delsys system. The explained variance when recording with the Myoware dropped to within 19 and 78%. Although ranges of explained variance suggest higher sensitivity to noise when using Myoware, similar regression slopes during force build-up and the distributions of ER values during static holds indicate that for our purposes, the two systems may provide similar measures of muscle activity.

### 3.2. Feasibility, Safety, and Adherence

**Figure 5:**
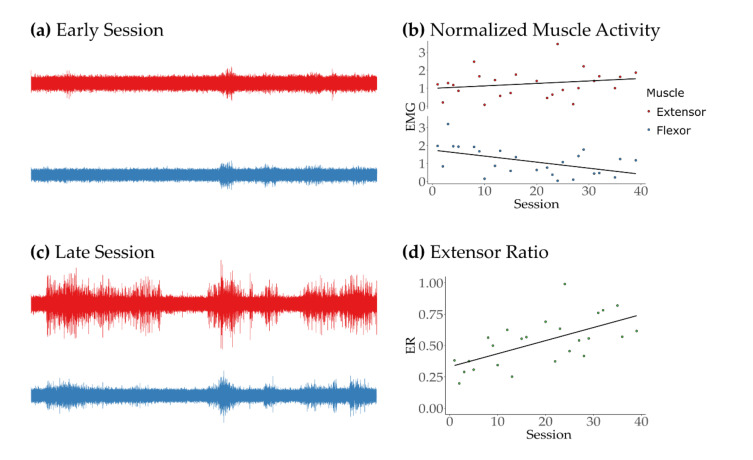
Muscle activity during REINVENT Sessions. Example of representative activity from the extensor (red) and flexor (blue) muscles during the calibration of early (**a**) and late (**c**) trials. The calibration algorithm looks at the recorded activity during which a video cued movement attempts and calculates the highest rectified muscle activity of a 250-ms moving window average for each muscle. These values are later used to normalize the activity during each trial and calculate extensor ratios (ER) to control the game. (**b**) Normalized muscle activity (EMG) averaged across 40 sessions for extensor and flexor muscles. Changes over time were not significant. (**d**) Calculated values of ER averaged across 40 sessions. A significant increase in extension individuation appeared during movement attempts (*p* < 0.01). Regression lines are shown for visualization in panels (**b**) and (**d**).

The participant with stroke used the system for 40 sessions over 10 weeks, with 100% adherence to the requested protocol. In terms of user experience, the participant reported no perceived discomfort, pain, or fatigue, and there were no adverse events during any sessions using the system. The average session duration was 40 min, including about 10 min for setup, 30 min of repeated practice (5 blocks of 20 trials per session), and 1–2 min of rest between blocks of trials. Qualitatively, after 40 sessions, values of calculated individuated activity of the extensor muscle showed a significant increase over time (rho = 0.59, *p* < 0.001), as shown in Figure 5d. Normalized activity for the extensor muscle appeared to increase over time, while flexor activity decreased, as seen in Figure 5b. However, these individual changes were not statistically significant for either the extensor (rho = 0.27, *p* = 0.164) or the flexor (rho = −0.34, *p* = 0.071) muscles. Similarly, while changes in game performance improved, this change did not reach statistical significance (rho = 0.29, *p* = 0.06). Finally, the participant reported positive changes in motor function, e.g., increased extension at the interphalangeal joints and decreased frequency of muscle spasms, as well as improved overall quality of life, e.g., improved quality and duration of sleep. The participant also reported enjoyment of using the system and requested to keep it after the testing period concluded.

## 4. Discussion

In this paper, we report the development of a muscle-computer interface to train muscle activity from wrist extensors while limiting unintended coactivation of wrist flexors for at-home chronic stroke telerehabilitation. We first described the hardware and software components of the system including acquisition, processing pipeline, and feedback in the form of game control. We also provided a validation example in two healthy individuals, comparing the use of low-cost sensors to calculate ratios of muscle activity versus research-grade sensors while performing the same task. Finally, we showed the preliminary feasibility, safety, adherence, and efficacy in a single case study of one person with a stroke using this system for 40 sessions over 10 weeks. Overall, although the data are limited, our preliminary results suggest that our low-cost, portable EMG biofeedback system may be used with telerehabilitation to contribute to the development of accessible technology to improve post-stroke recovery.

In a previous study, we showed that it is feasible to use a research-grade EMG acquisition system to train the activity of agonist muscles while avoiding the simultaneous activity of antagonists via EMG biofeedback [20]. Here, we showed that these ratios of muscle activity can be acquired in an individual with stroke using low-cost sensors. In line with previous work [24,34,35], we also show that using low-cost sensors can result in similar results to research-grade sensors for low forces during an isometric task in two healthy individuals, despite their differences regarding electrode type, in-board filtering, and sensitivity. However, further investigation with a larger population is required to ensure the validity and robustness of our system. Although a high proportion of the measured force variability during the ramp phase of our task was explained by the recorded muscle activity with either system, this proportion dropped when using the low-cost sensors. This was expected due to the lack of in-board filtering, making the sensor more susceptible to noise and adding variability to the measurement. The absence of in-board filtering could also increase crosstalk, lower signal to noise ratios, and lower sensitivity, affecting the quality of EMG signals. While these challenges can be addressed, to some extent, with proper skin preparation and adequate sensor placement, it may also be beneficial to explore novel signal processing techniques to account for the variability of these factors across sessions in future home-based interventions. Previous research has evaluated the use of low-cost sensors and shown that they can be used as an alternative to research-grade equipment in healthy populations [34,35]. In this pilot study, we explored the use of these sensors with a stroke survivor, however, further research with an adequate sample size is required to evaluate the reliability of these systems for additional users with movement impairments. We used an isometric task as validation because most of our target population is incapable of performing volitional movement. In addition, validation of non-isometric movement tasks with simultaneous recordings using both low-cost technology and research-grade equipment could further improve the performance and accessibility of the Tele-REINVENT system.

Finally, we showed that our system was feasible, safe, and enjoyable to for 40 1-h sessions over 10 weeks of training. Notably, the participant used the system at home without any in-person instruction or guidance, relying only on telerehabilitation video calls and emails with the clinician and research team. The participant reported high satisfaction with the system, demonstrated 100% adherence to the research protocol, and requested to keep the system afterward. The participant also demonstrated significantly increased extensor activity while decreasing flexor activity over time. We anticipate that improved results could be gained with an initial in-person evaluation and familiarization with the system, as well as improved sensor placement at the first visit with muscle palpation to identify optimal electrode placement. In addition to in-person training, the Tele-REINVENT system could be further improved with enhanced calibration algorithms that account for placement variability across sessions as well as processing pipelines to help personalize the biofeedback for each individual. For example, including a higher number of sensors would allow the use of algorithms to detect the best combination of sensors, similar to the work by Lobov et al. [24]. Finally, although we showed moderate improvements in coordinated muscle activity, that is, reduced antagonist activation during attempted agonist activation, our reinforcement paradigm could be enhanced with improved in-game feedback. For example, in the future, we may incorporate adaptive control thresholds to encourage more individuated activity on a personalized basis, in a similar fashion to our previous work [20].

### Limitations and Future Work

In terms of hardware, although we demonstrated the validity of using the proposed sensors, future work may incorporate wireless communication between the computer and the sensors to increase mobility and provide enhanced electrical insulation for the sensors. Additionally, we used a laptop computer with specifications that allowed adequate real-time processing of the EMG signals and simultaneous rendering of the feedback game, along with video conferencing. However, future iterations may use lower-cost hardware, such as a tablet or cellular phone, instead of a full laptop. In terms of software, even though we made significant efforts to develop a user-friendly system, it still required a minimum level of computer literacy for adequate setup and use. Therefore, to further increase the system’s portability and acceptability, it would be crucial to optimize the acquisition and processing pipelines as well as the graphical interfaces to provide feedback in mobile devices, such as cellular phones and tablets. Furthermore, the variability of sensor location across sessions and the level of impairment of the participants make it necessary to develop and test more robust calibration and processing algorithms that account for such variability. For example, normalizing the recorded activity with the maximum amplitude registered during any attempted movements, as opposed to specifically using an attempted grip, would allow system usage to extend to participants without the ability to grasp an object. Improvements in user interaction and calibration will ensure both an easier setup and increased in-session robustness, even for sessions with inadequate sensor placement. In addition, an expanded catalog of games could increase not only the acceptability of the system but the variety of tasks to train. Finally, it is important to note that while the purpose of this work was to demonstrate the feasibility of developing and deploying a portable muscle–computer interface for telerehabilitation, the current investigation only provides a description of the system, a limited comparison of the low-cost sensors with research grade sensors in two healthy individuals, and a single case study with one person with stroke. Further research with large and diverse populations is needed to examine the efficacy of this system for telerehabilitation services, along with pre- and post-intervention behavioral assessments to further evaluate the effects of this system in promoting functional recovery at both individual and group levels. In summary, this work shows that using low-cost technology for individuated muscle training to reduce unintended coactivation for supervised and unsupervised home-based telerehabilitation is feasible and may widen the development of meaningful and personalized chronic stroke rehabilitation.

## Figures and Tables

**Table 1:** Score likelihood for the *SkeeBall* game as a function of the calculated extensor ratio (ER).

ER Value	Points	Likelihood
0.9 < ER ≤ 0.99	30	100%
0.6 < ER ≤ 0.9	30	40%
	20	60%
0.4 < ER ≤ 0.6	20	40%
	10	60%
0.2 < ER ≤ 0.4	10	20%
	0	80%
ER ≤ 0.2	0	100%

**Table 2:** Sensor specifications.

Specification	Delsys Trigno Avanti	Tele-REINVENT
EMG sensors	4	2
Inter-electrode spacing	10 mm	20 mm
EMG sampling rate (max)	4370 sa/sec	2000 sa/sec
Resolution	16 bits	12 bits
In-board amplification	1000 V/V	500 V/V
In-board filter (max range)	Butterworth bandpass (10–850 Hz)	NA ^1^
Estimated price	USD 8400 ^2^	USD 150 ^3^

^1^ NA = not available. This sensor does not incorporate in-board filtering. ^2^ Price may vary since a quote from the vendor is required. This price included the proprietary acquisition system and software (Trigno Avanti Platform) and 4 Trigno Mini Sensors. ^3^ Estimated cost of the required components to build a Tele-REINVENT system, including a Teensy 4.0 board and 2 Myoware sensors. Both Delsys and Tele-REINVENT require a laptop computer (not included in these estimates).

**Table 3:** Muscle activity correlation with grip force while building-up to a sustained grip.

Participant	Muscle	Sensor	Slope	R^2^
1	Extensor	Delsys	1.54	0.940
1	Extensor	Myoware	1.77	0.784
1	Flexor	Delsys	1.53	0.795
1	Flexor	Myoware	1.11	0.543
2	Extensor	Delsys	1.46	0.935
2	Extensor	Myoware	1.64	0.339
2	Flexor	Delsys	1.69	0.885
2	Flexor	Myoware	1.46	0.193

## Data Availability

Experimental data are available in the GitHub repository “npnl/REINVENT_data” (https://github.com/npnl/REINVENT_data (accessed on 25 January 2021)).
